# Biochemical Biomarker Combinations in Human Traumatic Spinal Cord Injury: An Exploratory Study

**DOI:** 10.3390/jcm15197385

**Published:** 2026-09-23

**Authors:** Kristoffer A. Aagaard, Thea O. Wichmann, Claus V. B. Hviid, Mikkel M. Rasmussen

**Affiliations:** 1 Department of Neurosurgery, Aarhus University Hospital, Palle Juul-Jensens Boulevard 165, 8200 Aarhus N, Denmark; thewic@rm.dk (T.O.W.); mikkrasm@rm.dk (M.M.R.); 2 Department of Neurosurgery, The Neuroscience Centre, Copenhagen University Hospital, Rigshospitalet, Blegdamsvej 9, 2100 Copenhagen, Denmark; 3 Department of Clinical Medicine, Aarhus University, Palle Juul-Jensens Boulevard 82, 8200 Aarhus N, Denmark; 4 Department of Anaesthesiology and Intensive Care, Aalborg University Hospital, Hospitalsbyen 1, 9260 Gistrup, Denmark; 5 Department of Clinical Biochemistry, Aalborg University Hospital, Hospitalsbyen 1, 9260 Gistrup, Denmark; 6 Department of Clinical Medicine, Aalborg University, Selma Lagerløfs Vej 249, 9260 Gistrup, Denmark

**Keywords:** traumatic spinal cord injury, biomarkers, blood, cerebrospinal fluid

## Abstract

**Background/Objectives:** Early diagnosis and prognosis after traumatic spinal cord injury (TSCI) remain challenging, and single biochemical biomarkers have shown limited stand-alone utility. Combining different biomarkers may improve prediction. We explored combinations of biomarkers in blood and cerebrospinal fluid (CSF) and their association with injury severity and neurological outcome after TSCI. **Methods:** In this post hoc analysis of a previously reported prospective cohort, blood and CSF were collected from 15 patients with TSCI at a median of 13 h after injury. Biomarkers comprised 13 inflammatory/structural proteins measured by quantitative assays and an Olink Target 96 Inflammation panel (92 proteins). Injury severity was classified by the American Spinal Injury Association (ASIA) Motor Score (AMS) at admission (admission injury severity) and at follow-up (follow-up neurological status). LASSO regression with 1000 repetitions identified combinations associated with level-adjusted AMS. **Results:** Of the quantitative assay biomarkers, interferon-γ (IFN-γ), interleukin (IL)-6 and IL-8 in blood and IFN-γ and IL-10 in CSF were frequently selected for admission injury severity, while IL-6, IL-8 and glial fibrillary acidic protein (GFAP) in blood and IFN-γ in CSF were selected for follow-up neurological status. Olink panel biomarkers varied substantially: Thymic stromal lymphopoietin (TSLP), matrix metalloproteinase-10 (MMP-10) and IL-17A in blood and C-C motif chemokine 3 (CCL3) in CSF were selected in admission injury severity models, and CCL20 and TSLP in blood and CCL3 in CSF were selected for follow-up neurological status. Forcing admission injury severity into the follow-up models reduced internally cross-validated prediction error by approximately 29–33% across all panels, suggesting that much of the apparent biomarker signal reflected admission injury severity rather than independent prognostic information. Only GFAP in blood retained a consistent association with follow-up neurological status when accounting for admission injury severity. **Conclusions:** In this small exploratory study, blood-based combinations of IFN-γ, IL-8 and IL-6 and CSF-based combinations of IFN-γ and IL-10 were associated with admission injury severity. Only GFAP in blood showed consistent prognostic value beyond admission severity. Selection frequencies and cross-validated estimates are internal and hypothesis-generating, not evidence of clinical utility or generalizability; independent validation is required.

## 1. Introduction

Early diagnosis and prognosis after TSCI represent a great challenge. The best predictor of clinical neurological outcome is the injury severity evaluated by the American Spinal Injury Association (ASIA) impairment scale (AIS) [1,2]. Evaluation of initial injury severity is frequently hampered by several factors, including co-existing injuries, intubation and sedation [3], thereby impeding early diagnosis and prognosis. To individualize and improve diagnostics and prognostication, extensive research efforts have been put into the development of biochemical biomarkers that can supplement clinical neurological evaluation [4,5,6,7,8]. One of the most studied biochemical biomarkers is glial fibrillary acidic protein (GFAP) [9,10,11]. Although GFAP is associated with clinical outcome [4,9,12], its stand-alone clinical utility in TSCI is limited by concomitant brain injury [13]. Similar limitations apply to other well-studied biomarkers, including neuron specific enolase (NSE), S100 calcium-binding protein B (S100B) and neurofilament light chain (NfL) [4,11,13,14]. Combining biomarkers with different cellular origins in predictive models may provide comprehensive information about the injury, thereby enhancing the strength and reliability of potential associations. Prior studies have mainly investigated the association of single biomarkers with outcomes [9,12,14]. Only a few TSCI studies have investigated biomarker combinations, e.g., S100B, GFAP, IL-6, IL-8, monocyte chemoattractant protein-1 (MCP-1) and tau [6,15], and these findings have not been validated in independent cohorts and have not been translated into clinical practice. Our previous cohort studies characterized the inflammatory response and blood–spinal cord barrier integrity and examined single biomarkers in relation to AIS grade [9,16,17]. The present study addresses whether combinations of biomarkers, selected by penalized regression, are associated with a level-adjusted continuous measure of motor function. This exploratory study aims to investigate which biochemical biomarker combinations are associated with admission injury severity and with follow-up neurological status after TSCI.

## 2. Materials and Methods

### 2.1. Study Population

This is a post hoc, exploratory secondary analysis of a prospectively enrolled cohort reported in our previous publications [9,16,17]; the present study addresses a new question (biomarker combinations associated with a continuous motor outcome) and does not overlap with the primary biomarker or clinical outcome results of those studies. The original cohort was registered at ClinicalTrials.gov (reg. no. NCT03505463). Approvals were obtained from the Central Denmark Region Committees on Health Research Ethics (1-10-72-382-17) and the Danish Data Protection Agency (1-1602-754-17). The study population consisted of TSCI patients included at the Department of Neurosurgery at Aarhus University Hospital, Denmark, between 2018 and 2020. Written informed consent was obtained from all patients. Inclusion and exclusion criteria are described in the previous studies [9,16,17].

### 2.2. Data Collection

Patients had blood and CSF samples collected at three time-points [16], but only samples collected upon admission were used for this study. Blood samples were collected from an arterial line or a venipuncture, while CSF samples were collected by lumbar puncture. The first lumbar puncture was performed prior to surgery in nine patients and immediately after surgery in six patients. None of the patients was treated with immunosuppressants or had signs of infection upon sample collection. Samples were stored at −80 °C until batch analysis. For more details on sample collection, handling and storage, see our previous studies [16,17]. Patients had clinical neurological evaluation performed at three time-points according to AIS and ASIA Motor Score (AMS) [2], but only clinical neurological evaluation upon admission, i.e., the first time-point, and follow-up, i.e., the last time-point, was used for this analysis. Clinical neurological evaluation was conducted by trained physicians or trained physiotherapists.

### 2.3. Biochemical Analysis

Biochemical analysis was performed as described in our previous study [16] during the same study period. Immunoassay analyses were carried out at Aarhus University Hospital using the Roche Cobas 8000 Modular Analyzer (Roche Diagnostics International AG, Rotkreuz, Switzerland) for neuron-specific enolase (NSE) analysis, the Neurology 2-plex B assay (Quanterix, Billerica, MA, USA) read on the Simoa platform for glial fibrillary acidic protein (GFAP) and neurofilament light chain (NfL) analysis, and the V-PLEX Proinflammatory Panel 1 Human Kit (Meso Scale Discovery, Inc., Rockville, MD, USA) read on the MESO QuickPlex SQ 120 for analysis of interferon-γ (IFN-γ), interleukin (IL)-1β, IL-2, IL-4, IL-6, IL-8, IL-10, IL-12p70, IL-13 and tumor necrosis factor α. The NSE, GFAP/NfL and Meso Scale Discovery analyses are collectively referred to as quantitative assays throughout this paper, as they report absolute concentrations, in contrast to the Olink panel, which reports protein levels on a relative scale. Proteomic analysis was carried out by BioXpedia A/S using the Olink Target 96 Inflammation panel (Olink Proteomics AB, Uppsala, Sweden) [17]. All samples were processed on a single 96-well plate. Intra-plate normalization was performed by Olink Proteomics/BioXpedia A/S using internal extension and detection controls, according to the manufacturer’s standard protocol; as all samples were run on the same plate, no inter-plate bridging normalization was required.

### 2.4. Statistical Analysis

For statistical analysis an adjusted AMS was generated to account for the dependency of AMS on the neurological level of injury. The lowest possible AMS value was defined based on ASIA guidelines [2]. For high cervical injuries (C1–C4), complete tetraplegia corresponds to a minimum AMS of 0. The minimum values increase with lower injury levels, thus C5 = 6, C6 = 16, C7 = 26, C8 = 36, T1 = 46, and T2 or below = 50. The adjusted AMS was calculated as:

(1) $$Adjusted\;AMS=\frac{AMS_{actual}-AMS_{min}}{100-AMS_{min}}\times 100$$

Thereby we aim to express each patient’s motor score as the proportion of motor function retained above the theoretical minimum for their level of injury. Adjusted AMS was calculated for AMS evaluated at the first time-point (admission, reflecting admission injury severity) and the last time-point (follow-up, reflecting follow-up neurological status).

Statistical analyses were carried out in R (v4.5.1, macOS). Least Absolute Shrinkage and Selection Operator (LASSO) regression was applied using the glmnet package (v4.1-10). Separate runs were performed modeling level-adjusted AMS against admission injury severity and against follow-up neurological status. The repeated LASSO analysis was the primary analysis. Matrices were generated independently for blood and CSF. Olink panel and quantitative assay biomarkers were analyzed in separate matrices due to their fundamentally different scales and measurement units. Several mediators (e.g., IFN-γ, IL-6) were measured on both platforms, but as the quantitative assays report absolute concentrations and the Olink panel reports relative NPX values, the two analyses were not compared numerically and were modeled separately. Variables were standardized as numeric values before analysis. A sequence of 100 logarithmically spaced λ values (10^10^ to 10^−2^) defined the regularization path. LASSO models were fit with glmnet (x, y, alpha = 1, lambda = grid), where alpha = 1 specifies pure LASSO regularization. The optimal λ (lambda.min) was determined via 10-fold cross-validation using cv.glmnet, minimizing mean cross-validated error. The model was refit using the optimal λ. Predictors with non-zero coefficients were extracted. This procedure was repeated 1000 times for each combination, incrementing the random seed by one for each iteration. The selection frequency for each biomarker across the 1000 runs was recorded. Biomarkers selected in ≥80% of iterations were referred to as frequently selected. This threshold is arbitrary and was chosen to aid interpretation, and not as a measure of statistical significance. The 1000 repetitions quantify the stability of biomarker selection within this cohort under resampling of the cross-validation folds; they do not estimate population-level reproducibility. New cross-validation folds were generated at each iteration by incrementing the random seed. No biomarker values were missing in the analyzed admission samples; concentrations below the lower limit of detection were assigned half the limit value, as in the original cohort analyses [16,17]. The complete analysis code is available from the corresponding author on request. Univariable association tests and multivariable regression models are reported in the Supplementary Materials as descriptive support.

To address whether biomarker selection for follow-up neurological status reflects prognostic information beyond what is already known at admission, three sensitivity analyses were performed for the follow-up models. First, admission injury severity was forced into every repeated LASSO model as a mandatory, unpenalized covariate (penalty factor = 0), and selection frequencies for all other biomarkers were re-derived under this constraint. Second, the outcome was reformulated as raw follow-up motor score with neurological level (represented by the theoretical minimum AMS for that level) forced in as a mandatory covariate, instead of being built into the outcome transformation; median cross-validated root mean squared error (RMSE) and the direction and magnitude of biomarker coefficients were recorded across the 1000 repetitions under this formulation. Third, biomarkers were classified by comparing their selection frequency across the original, baseline-adjusted, and outcome-reformulated analyses, using the same ≥80% threshold applied elsewhere in this manuscript. Biomarkers reaching ≥80% under all three formulations were classified as consistent; under exactly two formulations as robust (2/3); under only the original, unadjusted formulation as fragile, consistent with reflecting baseline severity rather than independent prognostic value; and under only one of the two sensitivity formulations as sensitivity-only. Only biomarkers reaching ≥80% under at least one formulation are reported.

Anthropic Claude was used for copywriting and spellchecking the manuscript and the Supplementary Materials, and for table formatting. It was not used for data analysis or interpretation.

## 3. Results

### 3.1. Study Population

A total of 15 patients with TSCI were included. Eleven patients had cervical injuries and four had thoracic injuries. Clinical neurological evaluation was performed at median 13 h (IQR: 19) and median 148 days (IQR: 49) after TSCI. Median admission AMS was 35 (IQR: 34.5) and median follow-up AMS was 54 (IQR: 44). The AIS grade distribution and detailed injury levels are reported in Supplementary Table S1.

A single patient had a reported AMS below the calculated minimum for his ASIA injury level, and in this case the AMS was readjusted to the lowest possible for his level of injury (from 48 on initial evaluation and 46 at follow-up adjusted to 50 in a patient with a low thoracic injury).

Eight combinations of biomarkers and outcome were analyzed with 1000 LASSO runs. The frequency of biomarker selection is shown for the quantitative assays (Supplementary Tables S14 and S15) and the Olink panel (Supplementary Tables S16 and S17).

### 3.2. Quantitative Assay Biomarkers

The admission injury severity models showed that IFN-γ and IL-10 were frequently selected in CSF (Figure 1).

Both GFAP and NfL appeared sporadically in CSF models. IFN-γ, IL-6, and IL-8 were frequently selected in blood (Supplementary Table S14). The follow-up neurological status models showed that IL-6, IL-8 and GFAP were frequently selected in the blood. In CSF, only IFN-γ was selected in all models. GFAP and IL-10 appeared with low frequency in CSF models (Supplementary Table S15).

### 3.3. Olink Panel Biomarkers

The admission injury severity models showed that TSLP, MMP-10 and IL-17A were frequently selected in blood, while CD8A, CCL20, CCL3, IL-24 and IL-2 were selected less often. Multiple CSF biomarkers were included in the admission injury severity models with CCL3 included in all models. Biomarkers including TWEAK, CX3CL1, IFN-γ, CCL19, LIF-R and IL-6 were selected at lower frequencies (Supplementary Table S16). In the follow-up neurological status models, CCL20 and TSLP in blood were selected in all models. In CSF, only CCL3 was selected in all models, while multiple biomarkers including IFN-γ, IL-20RA, FGF-5, CCL28, CCL4, CD40, CDCP1, CXCL9 and IL-12B, were frequently selected (Supplementary Table S17).

### 3.4. Sensitivity Analyses: Robustness of Follow-Up Associations

To evaluate whether the follow-up associations reflected prognostic information beyond admission severity, biomarker selection was compared across the original, baseline-adjusted, and outcome-reformulated formulations (Table 1).

In blood, only GFAP remained selected at high frequency across all three formulations, suggesting that it was the only biomarker carrying prognostic information independent of admission injury severity. IFN-γ in CSF, IL-4 in CSF, TSLP in blood, and CXCL9 in CSF were selected at high frequency in two of the three formulations but were not selected, or were selected only sporadically, under the third. The remaining biomarkers previously identified as frequently selected for follow-up neurological status, including IL-8 and IL-6 in blood, as well as CCL3, CCL20, IL-20RA, FGF-5, CCL28, CCL4, CD40, CDCP1, and IL-12B, were no longer selected once admission injury severity or neurological level was accounted for. This suggests that these selections reflect baseline injury severity rather than independent prognostic information. Median cross-validated RMSE for the outcome-reformulated models ranged from 28.0 to 32.0 points on the raw 0–100 AMS scale across the four panels (Supplementary Table S18), indicating a broadly comparable predictive uncertainty regardless of sample type or platform.

## 4. Discussion

This exploratory study identified blood- and CSF-based biomarker combinations associated with admission injury severity and with follow-up neurological status after TSCI. Our main findings suggest associations for blood-based quantitative assay combinations of IFN-γ, IL-8 and IL-6 for admission injury severity. Additionally, we identified CSF-based quantitative assay combinations of IFN-γ and IL-10 for admission injury severity. When accounting for admission injury severity, only GFAP in blood retained a consistent association with follow-up neurological status. Olink panel associations showed substantial variability with many biomarkers appearing at different frequencies.

Identifying biomarker combinations associated with clinical neurological outcome is highly warranted as an additive to improve diagnosis and prognosis after TSCI. Several studies have reported associations between biochemical biomarkers and clinical outcome after TSCI as a supplement to clinical neurological evaluation [4,5,6,7,8]. In our exploratory study, we explored quantitative assay and Olink panel biomarkers in blood and CSF. Using this dataset, our previous study found IFN-γ and GFAP in blood and GFAP, IFN-γ and IL-10 in CSF to be associated with AIS grade upon admission [9]. This highlights that biomarker selection depends on the outcome measure of interest.

The CSF-based quantitative assay models of admission injury severity suggest that a combination of IFN-γ and IL-10 could be a candidate for further study. GFAP is a frequently studied biomarker in TSCI, and studies report that GFAP in CSF correlates with injury severity [4,9], while NfL in CSF has only been sparsely studied. Both GFAP and NfL appear in a few of the CSF models generated, raising questions about the external validity of the models generated in our analysis. The repeated LASSO and univariable analyses do not always agree. This is expected, as the two approaches answer different questions: univariable testing evaluates each marker in isolation, whereas penalized regression retains a marker only when it adds information beyond the other co-modeled markers. These results should, therefore, be read as complementary rather than contradictory. The blood-based quantitative assay biomarkers of follow-up neurological status showed a consistent pattern of selecting IL-8, GFAP, and IL-6. However, sensitivity analyses forcing admission injury severity or neurological level into the models showed that only GFAP remained robustly selected; IL-8 and IL-6 were no longer selected once baseline severity was accounted for. This indicates that the apparent prognostic value of IL-8 and IL-6 for follow-up neurological status largely reflects their correlation with admission severity rather than independent prognostic information, whereas GFAP shows more consistent evidence of carrying information beyond what is already known at admission. Consistent with this, forcing admission severity into the follow-up models reduced cross-validated prediction error by roughly one third across all panels, indicating that admission severity accounted for most of the achievable predictive information.

The Olink panel biomarker models of admission injury severity showed substantial variability and varying frequencies, suggesting most associations are relatively weak. TSLP was consistently selected in blood. TSLP is expressed in the central nervous system, e.g., by astrocytes in the spinal cord [18], but has not previously been examined as a biomarker in TSCI. In CSF, TSLP was not selected as frequently, while CCL3 and TWEAK were included in most models. TWEAK has not been examined as a biomarker of injury severity in TSCI, but heightened signaling has been found in the paralyzed muscles of men with chronic TSCI [19]. CCL3 has in mice been found to contribute to the secondary damage after SCI [20,21]; these are experimental findings in animal models, and neither mechanistic involvement in human TSCI nor a role as a biomarker of injury severity has been established.

CCL3, CCL20, and MMP-10 were among the biomarkers most frequently selected in the present analysis; notably, in a previous analysis of this same cohort using AIS grade as the outcome measure, CCL3, CCL20, and related proteins were similarly associated with admission injury severity [17]. The present combination analysis therefore partly re-expresses previously reported single-marker associations within the same 15 patients rather than identifying wholly new signals, and the sensitivity analyses in Section 3.4 indicate that these particular associations largely reflect admission severity.

The Olink panel biomarker models of follow-up neurological status showed stable selection of CCL20 and TSLP in blood. CCL20 has been reported to aggravate neuroinflammation after TSCI in rats [22]; whether it plays a comparable role in human TSCI is unknown. However, when admission injury severity was forced into the model as a covariate, neither CCL20 nor TSLP was selected; TSLP was again selected under the outcome-reformulated analysis, whereas CCL20 was not. These biomarkers should therefore be interpreted with caution as candidates carrying outcome-specific rather than severity-related information. In CSF, IFN-γ, FGF-5, CCL28, CDCP1, IL-12B, IL-20RA, CD8A, CCL4, CD40, and CXCL9 were frequently co-selected. Given the small sample size and the large number of correlated proteins, this co-selection most likely reflects collinearity rather than independent associations. Of these, only CXCL9 remained selected at high frequency after forcing admission injury severity into the model, though it was not selected under the alternative outcome-reformulated analysis; the remaining proteins were no longer selected under either sensitivity analysis, consistent with their original selection reflecting shared correlation with admission severity rather than independent prognostic signal.

This analysis did not include a control group for comparison. The original studies included a control group; however, this study explored biomarkers within injured patients rather than testing whether biomarkers discriminate TSCI from non-injured individuals, and this severity-stratification design does not rely on a non-injured reference.

Realistic clinical implementation of biomarker combinations of these kinds would require a defined translational pathway. Given their exploratory selection here, these combinations are more plausible as adjuncts to standardized clinical neurological evaluation, for instance, by refining early risk stratification when ASIA assessment is confounded by sedation, intubation, or concomitant injury, rather than as stand-alone diagnostic or prognostic tools. Moving toward clinical use would require rapid, standardized assay platforms with defined reference ranges, prospective validation of fixed, rather than data-driven, biomarker panels in independent cohorts, and evidence that the added biomarker information changes clinical management or prognostic counseling beyond standard neurological evaluation alone. Until these steps are completed, the combinations identified in this exploratory analysis should be regarded as hypothesis-generating candidates rather than clinically actionable panels.

### Strengths and Limitations

This study has notable strengths. Paired blood and CSF sampling from the same patients allowed biomarker selection to be compared across compartments, which few TSCI biomarker studies have achieved, and the prospectively enrolled cohort was well characterized, with standardized clinical evaluation and defined sampling intervals. Biomarkers were assessed across two complementary platforms: established quantitative assays and a broad Olink inflammation panel providing wide biomarker coverage.

Some limitations also need to be addressed. The most important limitation is the combination of a very small sample (*n* = 15) and a very large number of candidate biomarkers (13 quantitative assays and 92 Olink panel proteins in two compartments), which carries a substantial risk of type I errors and of overfitting. To reduce overinterpretation, we used repeated LASSO modeling and focused on selection stability rather than individual model estimates. Statistical power and generalizability are therefore limited. Given the cohort size, a sensitivity analysis restricting the LASSO models to preoperatively sampled patients alone (*n* = 9) was not feasible. As CSF IL-6 has previously been associated with perioperative timing in this cohort [16], findings involving IL-6 should be interpreted with this potential confounder in mind. Blood was obtained from either an arterial line or venipuncture, and the sampling route was not tested as a covariate; it should be regarded as an unmeasured preanalytical source of variation for the blood-based findings. This emphasizes the exploratory nature of the study.

Protein expression in the Olink analysis was measured as normalized protein expression (NPX), a relative quantification unit provided by the manufacturer [23]. NPX values cannot be directly compared across runs without normalization, and although they are logarithmically related to protein concentration, they do not correspond to absolute concentrations in blood or CSF [23]. As a result, associations identified from Olink data require external validation under a harmonized assay and preprocessing protocol before they can be considered transferable to other sample runs; this reflects a need for external calibration rather than an intrinsic limitation of NPX-based prediction.

The use of AMS as an outcome measure is objective, with a high interrater correlation [24]. Myotome selection is intended to determine the neurological level of injury rather than to predict functional outcomes [25]. We constructed a level-adjusted AMS score for this study to account for differences in injury level. This adjusted score is a study-specific normalization developed for this exploratory analysis, and it has not been independently validated and should not be regarded as an established outcome measure; however, splitting AMS into upper and lower extremity scores may better reflect functional differences [25].

Finally, the volume of damaged tissue and the severity of clinical deficit are not always concordant, as deficit severity depends on which tracts are involved rather than on lesion volume alone [26]. Correlating biomarker levels with the extent of cord signal change on MRI would help disentangle these effects and is an important direction for future work. Combining plasma biomarkers with quantitative MRI measures has been shown to improve prediction of neurological outcome in cervical TSCI [27,28].

## 5. Conclusions

This exploratory study identified blood- and CSF-based biomarker combinations associated with admission injury severity and follow-up neurological status in 15 patients with TSCI. Our study suggests that blood-based combinations of IFN-γ, IL-8 and IL-6, and CSF-based combinations of IFN-γ and IL-10 were associated with injury severity at admission. For follow-up neurological status, GFAP in blood was the only biomarker consistently associated with outcome beyond admission injury severity. Given the small sample, these selection frequencies and cross-validated estimates are internal and hypothesis-generating, and should not be interpreted as evidence of clinical utility or generalizability; independent validation in larger cohorts is required.

## Figures and Tables

**Figure 1:**
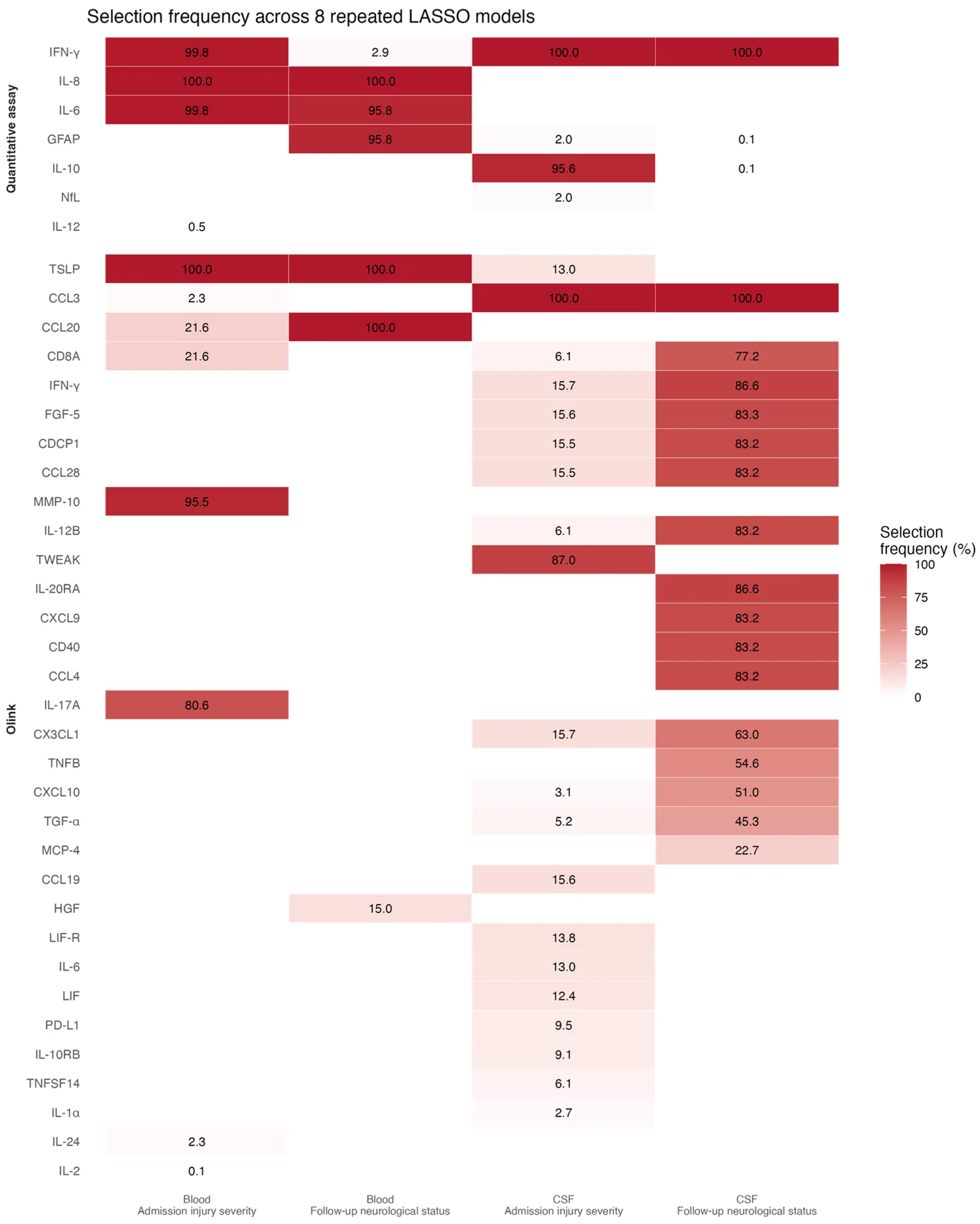
Selection frequency across all eight combinations of biomarker sets and outcomes.

**Table 1:** Robustness of biomarker selection for follow-up neurological status across three analytical formulations.

Panel	Sample Type	Biomarker	Original (%)	Adjusted (%)	Reform. (%)	Direction	Verdict
Quant.	Blood	GFAP	95.8	100.0	100.0	−	Consistent
Quant.	CSF	IFN-γ	100.0	100.0	0.0	−	Robust (2/3)
Quant.	CSF	GFAP	0.1	0.0	100.0	−	Sensitivity-only
Quant.	CSF	IL-4	0.0	91.1	66.1	−	Sensitivity-only
Quant.	CSF	NSE	0.0	0.0	96.0	−	Sensitivity-only
Quant.	Blood	IL-8	100.0	0.0	0.0	−	Fragile
Quant.	Blood	IL-6	95.8	0.0	0.0	−	Fragile
Olink	Blood	TSLP	100.0	0.0	100.0	−	Robust (2/3)
Olink	CSF	CXCL9	83.2	89.7	0.0	−	Robust (2/3)
Olink	CSF	MCP-4	22.7	0.0	98.4	−	Sensitivity-only
Olink	Blood	GDNF	0.0	100.0	3.5	+	Sensitivity-only
Olink	Blood	SCF	0.0	0.1	100.0	+	Sensitivity-only
Olink	CSF	IL-10RA	0.0	89.7	0.0	−	Sensitivity-only
Olink	CSF	HGF	0.0	89.4	0.7	−	Sensitivity-only
Olink	CSF	TNF-α	0.0	80.5	0.0	−	Sensitivity-only
Olink	Blood	CCL20	100.0	0.0	37.6	−	Fragile
Olink	CSF	CCL3	100.0	0.0	1.8	−	Fragile
Olink	CSF	IFN-γ	86.6	8.4	2.0	MIXED	Fragile
Olink	CSF	IL-20RA	86.6	11.1	2.0	+	Fragile
Olink	CSF	FGF-5	83.3	0.0	2.4	+	Fragile
Olink	CSF	CCL28	83.2	11.1	1.7	+	Fragile
Olink	CSF	CCL4	83.2	23.4	0.0	−	Fragile
Olink	CSF	CD40	83.2	0.0	1.7	−	Fragile
Olink	CSF	CDCP1	83.2	0.0	1.9	+	Fragile
Olink	CSF	IL-12B	83.2	0.0	1.9	−	Fragile

Table 1: Selection frequency (%) across 1000 repeated LASSO models predicting follow-up neurological status, under three formulations: the original level-adjusted percentage outcome (Original); the same outcome with admission injury severity forced into every model as a mandatory covariate (Adjusted); and raw follow-up motor score with neurological level forced in as a covariate instead of being built into the outcome transformation (Reform). Only biomarkers selected in ≥80% of models under at least one formulation are shown; the same threshold used to define “frequently selected” elsewhere in this manuscript. Direction: sign of the median coefficient in the formulation(s) where the biomarker was selected (+ positive, − negative, and MIXED indicates the sign differed between formulations). Verdict: Consistent: selected at ≥80% under all three formulations. Robust (2/3): ≥80% under exactly two formulations. Fragile: ≥80% only in the original, unadjusted formulation, consistent with reflecting baseline severity rather than independent prognostic value. Sensitivity-only: ≥80% only after adjustment or reformulation, not in the original analysis.

## Data Availability

The datasets generated and analyzed during the current study are not publicly available because of restrictions imposed by the Danish Data Protection Agency and the Central Denmark Region Committees on Health Research Ethics to protect participant privacy but are available from the corresponding author on reasonable request and subject to the relevant approvals.

## Supplementary Materials

The following supporting information can be downloaded at: https://www.mdpi.com/article/10.3390/jcm15197385/s1, Table S1. Demographics of the study population; Table S2. ANOVA peripheral blood quantitative assay biomarkers; Table S3. ANOVA peripheral blood Olink panel biomarkers; Table S4. ANOVA cerebrospinal fluid quantitative assay biomarkers; Table S5. ANOVA cerebrospinal fluid Olink panel biomarkers; Table S6. Multiple linear regression based on the selected quantitative assay biomarkers in peripheral blood against admission injury severity; Table S7. Multiple linear regression based on the selected quantitative assay biomarkers in peripheral blood against the follow-up neurological status; Table S8. Multiple linear regression based on the selected Olink panel biomarkers in peripheral blood against admission injury severity; Table S9. Multiple linear regression based on the selected Olink panel biomarkers in peripheral blood against the follow-up neurological status; Table S10. Multiple linear regression based on the selected quantitative assay biomarkers in cerebrospinal fluid against the follow-up neurological status; Table S11. Multiple linear regression based on the selected quantitative assay biomarkers in cerebrospinal fluid against admission injury severity; Table S12. Multiple linear regression based on the selected Olink panel biomarkers in cerebrospinal fluid against admission injury severity; Table S13. Multiple linear regression based on the selected Olink panel biomarkers in cerebrospinal fluid against the follow-up neurological status; Table S14. Selection frequency of quantitative assay biomarkers modeling admission injury severity in repeated LASSO models; Table S15. Selection frequency of quantitative assay biomarkers modeling follow-up neurological status in repeated LASSO models; Table S16. Selection frequency of Olink panel biomarkers modeling admission injury severity in repeated LASSO models; Table S17. Selection frequency of Olink panel biomarkers modeling follow-up neurological status in repeated LASSO models; Table S18. Cross-validated predictive performance of repeated LASSO models.

## Abbreviations

The following abbreviations are used in this manuscript:

TSCI	Traumatic spinal cord injury
CSF	Cerebrospinal fluid
ASIA	American Spinal Injury Association
AMS	ASIA Motor Score
AIS	ASIA Impairment Scale
MRI	Magnetic resonance imaging
GFAP	Glial fibrillary acidic protein
NfL	Neurofilament light chain
NSE	Neuron-specific enolase
S100B	S100 calcium-binding protein B
IFN-γ	Interferon-γ
IL	Interleukin
TSLP	Thymic stromal lymphopoietin
MMP-10	Matrix metalloproteinase-10
CCL3	C-C motif chemokine 3
NPX	Normalized protein expression
LASSO	Least absolute shrinkage and selection operator
IQR	Interquartile range
RMSE	Root mean squared error
CCL20	C-C motif chemokine 20
CXCL9	C-X-C motif chemokine 9
TWEAK	TNF-related weak inducer of apoptosis
IL-10RA	Interleukin-10 receptor subunit alpha
GDNF	Glial cell line-derived neurotrophic factor
SCF	Stem cell factor
HGF	Hepatocyte growth factor
MCP-1	Monocyte chemoattractant protein-1
TNF-α	Tumor necrosis factor-alpha
