# Supplementary material for: Biochemical Biomarker Combinations in Human Traumatic Spinal Cord Injury: An Exploratory Study
Source: J Clin Med. 2026 Sep 23;15(19):7385. doi: 10.3390/jcm15197385 (PMC13665330; doi:10.3390/jcm15197385)
Supplement: Supplementary file 1 [file jcm-15-07385-s001.zip › jcm-4529323-supplementary.pdf]

Methods

Study population

In addition to the TSCI cohort described in the main article, 15 non-TSCI patients scheduled for elective hip surgery at the Department of Orthopedic Surgery, Aarhus University Hospital, Denmark, were recruited in 2020 and included in this analysis.

Data collection

Biological samples, including peripheral blood (PB) and cerebrospinal fluid (CSF), were collected from all participants. In the TSCI group, analysis and follow up was performed as described in the main article. For non-TSCI controls, PB and CSF were collected at a single time point.

Statistical analysis

In order to reduce the number of independent variables included in the linear regression models, a two-step selection strategy was applied. First, one-way analysis of variance (ANOVA) was conducted separately for each biomarker, with acute AIS grade as the dependent variable. To strengthen discriminatory power, non-TSCI patients were included in the analysis as a distinct comparison group, thereby broadening the variance across which potential biomarker differences could be evaluated. Biomarkers demonstrating statistically significant group differences ($p \leq 0.05$) were identified from both peripheral blood (PB) and cerebrospinal fluid (CSF) datasets, encompassing both quantitative assay and Olink sources. This filtering step served to exclude variables unlikely to carry meaningful associations with injury severity.

The subset of biomarkers identified through ANOVA was subsequently incorporated into multiple linear regression models, with the adjusted American Spinal Injury Association (ASIA) Motor Score (AMS) values at both the admission and follow-up time points serving as the dependent variables. This approach allowed for an initial assessment of biomarker combinations that may explain variation in functional outcomes while minimizing the risk of overfitting that would arise from including the full set of candidate biomarkers. Although exploratory in nature, this stepwise reduction strategy provided a pragmatic balance between retaining potentially informative variables and maintaining model parsimony.

Results

Demographics

Table S1. Demographics of the study population

	**TSCI patients**	**Non-TSCI patients**[a]
Count, *n*	15	15
**Sex, *n* (%)**		

	TSCI patients	Non-TSCI patients[a]
Women	4 (27%)	7 (47%)
Men	11 (73%)	8 (53%)
Age, mean year (range)	50 (21–75)	51 (18–82)
**Follow-up, median days (IQR)**		
Acute	0 (1)	
Subacute	9 (2)	
Chronic	148 (49)	
**Mechanism of injury, *n* (%)**		
Fall	7 (47%)	
Traffic accident	7 (47%)	
Sports	1 (7%)	
**Level of injury, *n* (%)**		
Cervical	11 (73%)	
Thoracic	4 (27%)	
Lumbar	0 (0%)	
**AIS grade, *n* (%)**		
A	6 (40%)	
B	2 (13%)	
C	4 (27%)	
D	3 (20%)	
**AIS grade conversion, *n* (%)[b]**		
Yes	4 (36%)	
No	7 (64%)	

Table S1. Demographics of the study population. Values are n (%) unless otherwise indicated. Follow-up time, mechanism of injury, level of injury, AIS grade, and AIS grade conversion are shown for the TSCI group only; these characteristics correspond to the cohort described in our previous studies of this material. AIS: American Spinal Injury Association Impairment Scale; IQR: interquartile range; TSCI: traumatic spinal cord injury. [a] Non-TSCI refers to the reference group of patients without spinal cord injury. [b] AIS grade conversion could be assessed in the eleven TSCI patients with complete follow-up.

ANOVA

Table S2. ANOVA peripheral blood quantitative assay biomarkers

Predictor	P Value	η2
IL-6	0.0097**	0.402
IL-8	0.0102*	0.399
IFN-γ	0.2454	0.189
TNF-α	0.2669	0.182
IL-2	0.9758	0.018
IL-12	0.9660	0.022
IL-4	0.7484	0.072
IL-10	0.1377	0.235
IL-13	0.6330	0.094
NfL	0.0091**	0.406
GFAP	0.0013**	0.497

Table S2. One-way ANOVA results for quantitative assay peripheral blood biomarkers in TSCI and non-TSCI patients. The analysis was performed with AIS grade as the dependent variable, including non-TSCI patients as a separate comparison group. P values and effect sizes (η^2) are reported. IL-6, IL-8, NfL, and GFAP showed nominally significant group differences. As these are univariate, uncorrected comparisons in a small sample, they are reported for descriptive screening only and should not be interpreted as evidence of associations with injury severity.

Table S3. ANOVA peripheral blood Olink panel biomarkers

Predictor	P Value	η2
IL-8	0.0007***	0.523
VEGFA	0.0175*	0.370
CD8A	0.0637	0.290
MCP-3	0.0060**	0.427
GDNF	0.0473*	0.310
CDCP1	0.4648	0.129
CD244	0.6292	0.095
IL-7	0.1030	0.257
OPG	0.7603	0.069
LAP-TGF-β-1	0.2788	0.178
uPA	0.0238*	0.352

Predictor	P Value	η2
IL-6	0.0016**	0.488
IL-17C	0.5620	0.108
MCP-1	0.3442	0.159
IL-17A	0.2964	0.172
CXCL11	0.1240	0.243
AXIN1	0.4059	0.143
TRAIL	0.0283*	0.342
IL-20RA	0.0239*	0.352
CXCL9	0.4568	0.131
CST5	0.7472	0.072
OSM	0.1552	0.226
IL-2	0.3035	0.170
CXCL1	0.4448	0.134
TSLP	0.0279*	0.343
CCL4	0.1745	0.217
CD6	0.1162	0.248
SCF	0.0118*	0.392
IL-18	0.3822	0.149
SLAMF1	0.5139	0.118
TGF-α	0.4991	0.121
MCP-4	0.1915	0.210
CCL11	0.0033**	0.456
TNFSF14	0.0129*	0.387
FGF-23	0.1202	0.246
IL-10RA	0.1372	0.236
FGF-5	0.1477	0.230
MMP-1	0.2470	0.188
LIF-R	0.1191	0.246
FGF-21	0.2023	0.205
CCL19	0.0201*	0.362

Predictor	P Value	η2
IL-15RA	0.1981	0.207
IL-10RB	0.0909	0.266
IL-22-RA1	0.4377	0.135
IL-18R1	0.4387	0.135
PD-L1	0.2801	0.177
CXCL5	0.0414*	0.318
TRANCE	0.0004***	0.551
HGF	0.1180	0.247
IL-12B	0.1408	0.234
IL-24	0.1283	0.241
ARTN	0.3793	0.149
MMP-10	0.1427	0.233
IL-10	<0.0001***	0.656
TNF-α	0.4175	0.140
CCL23	0.0059**	0.428
CD5	0.2489	0.188
CCL3	0.0363*	0.327
Flt3L	0.0036**	0.452
CXCL6	0.5549	0.110
CXCL10	0.2086	0.203
4E-BP1	0.3006	0.171
IL-20	0.1208	0.245
SIRT2	0.5044	0.120
CCL28	0.0810	0.274
DNER	0.1995	0.206
EN-RAGE	0.0002***	0.567
CD40	0.5146	0.118
IL-33	0.0180*	0.368
IFN-γ	0.2462	0.189
FGF-19	0.1474	0.230

Predictor	P Value	η2
IL-4	0.2527	0.186
LIF	0.1883	0.211
NRTN	0.6206	0.096
MCP-2	0.0661	0.288
CASP-8	0.4837	0.125
CCL25	0.0475*	0.310
CX3CL1	0.0182*	0.368
TNFRSF9	0.4800	0.126
NT-3	0.1119	0.251
TWEAK	0.0412*	0.319
CCL20	0.0457*	0.312
ST1A1	0.2876	0.175
STAMBP	0.5451	0.112
IL-5	0.7188	0.077
ADA	0.5089	0.119
TNFB	0.0444*	0.314
CSF-1	0.1923	0.209

Table S3. One-way ANOVA results for Olink panel peripheral blood biomarkers in TSCI and non-TSCI patients. The analysis was performed with AIS grade as the dependent variable, including non-TSCI patients as a separate comparison group. P values and effect sizes (η^2) are reported. Multiple biomarkers, including IL-8, IL-6, IL-10, CCL11, TRANCE, EN-RAGE, and Flt3L, showed nominally significant group differences. Additional biomarkers such as VEGFA, MCP-3, uPA, TSLP, SCF, TNFSF14, CCL19, CXCL5, CCL23, CCL3, IL-33, CX3CL1, TWEAK, CCL20, and TNFB also reached nominal significance. Because 87 proteins were tested in 15 patients without correction for multiple comparisons, these results are highly susceptible to false-positive findings and are reported for descriptive screening only, not as evidence of robust associations or as a basis for outcome prediction.

Table S4. ANOVA cerebrospinal fluid quantitative assay biomarkers

Predictor	P Value	η2
IL-6	0.0317*	0.335
IL-8	0.0005***	0.535
IFN-γ	<0.0001***	0.663

Predictor	P Value	η2
TNF-α	0.0003***	0.561
IL-2	0.0007***	0.523
IL-12	0.0005***	0.535
IL-4	0.0009***	0.514
IL-10	<0.0001***	0.626
IL-13	<0.0001***	0.622
NSE	0.0029**	0.476
NfL	0.0650	0.289
GFAP	0.0013**	0.497

Table S4. One-way ANOVA results for quantitative assay CSF biomarkers in TSCI and non-TSCI patients. The analysis was performed with AIS grade as the dependent variable, including non-TSCI patients as a separate comparison group. P values and effect sizes (η^2) are reported. Several biomarkers, including IL-8, IFN-γ, TNF-α, IL-2, IL-12, IL-4, IL-10, IL-13, NSE, GFAP, and IL-6, showed nominally significant group differences, while NfL did not. As these are univariate, uncorrected comparisons in a small sample, they are reported for descriptive screening only and should not be interpreted as evidence of associations with injury severity or as predictors of functional outcome after TSCI.

Table S5. ANOVA cerebrospinal fluid Olink panel biomarkers

Predictor	P Value	η2
IL-8	<0.0001***	0.864
VEGFA	0.0038**	0.449
CD8A	0.7230	0.077
MCP-3	<0.0001***	0.724
CDCP1	0.2273	0.195
CD244	0.0067**	0.421
IL-7	0.1075	0.254
OPG	0.0027**	0.465
LAP-TGF-β-1	0.0228*	0.355
uPA	0.0031**	0.460
IL-6	<0.0001***	0.830
IL-17C	0.0596	0.295

Predictor	P Value	η2
MCP-1	<0.0001***	0.774
IL-17A	0.1925	0.209
CXCL11	0.0031**	0.460
AXIN1	0.0016**	0.489
TRAIL	0.0107*	0.397
IL-20RA	0.1332	0.238
CXCL9	0.0297*	0.339
CST5	0.0332*	0.332
IL-1α	0.0006***	0.528
OSM	<0.0001***	0.834
CXCL1	<0.0001***	0.771
TSLP	0.0082**	0.411
CCL4	<0.0001***	0.671
CD6	0.0419*	0.318
SCF	0.0830	0.272
IL-18	<0.0001***	0.682
TGF-α	0.1312	0.239
MCP-4	0.0006***	0.531
CCL11	0.0010***	0.510
TNFSF14	0.0052**	0.434
FGF-23	0.4981	0.122
IL-10RA	0.0555	0.299
FGF-5	0.0004***	0.547
MMP-1	0.0001***	0.585
LIF-R	0.1570	0.225
FGF-21	0.0239*	0.352
CCL19	0.0404*	0.320
IL-15RA	0.1121	0.251
IL-10RB	0.2379	0.192
IL-18R1	0.0266*	0.346

Predictor	P Value	η2
PD-L1	0.0998	0.259
CXCL5	0.0001***	0.598
HGF	0.1330	0.238
IL-12B	0.0996	0.259
ARTN	0.3207	0.165
MMP-10	0.0129*	0.387
IL-10	<0.0001***	0.798
TNF-α	<0.0001***	0.640
CCL23	0.0462*	0.311
CD5	0.0018**	0.484
CCL3	<0.0001***	0.730
Flt3L	0.0383*	0.323
CXCL6	0.0001***	0.591
CXCL10	0.0018**	0.483
4E-BP1	<0.0001***	0.631
IL-20	0.0100*	0.400
SIRT2	0.0019**	0.481
CCL28	0.0320*	0.334
DNER	0.0315*	0.335
EN-RAGE	0.0188*	0.366
CD40	0.1063	0.255
IL-33	0.0048**	0.438
IFN-γ	0.0077**	0.414
FGF-19	0.0529	0.302
IL-4	0.4664	0.129
LIF	<0.0001***	0.821
MCP-2	<0.0001***	0.748
CASP-8	<0.0001***	0.715
CCL25	0.1284	0.241
CX3CL1	0.2967	0.172

Predictor	P Value	η2
TNFRSF9	0.0007***	0.524
NT-3	0.0268*	0.345
TWEAK	0.0015**	0.492
CCL20	<0.0001***	0.768
ST1A1	0.0055**	0.432
STAMBP	<0.0001***	0.704
ADA	0.0146*	0.380
TNFB	0.6362	0.093
CSF-1	0.0004***	0.544

Table S5. One-way ANOVA results for Olink panel CSF biomarkers in TSCI and non-TSCI patients. The analysis was performed with AIS grade as the dependent variable, including non-TSCI patients as a separate comparison group. P values and effect sizes (η^2) are reported. Multiple biomarkers demonstrated statistically significant differences between groups, including IL-8, MCP-3, IL-6, OSM, CXCL1, CCL4, IL-18, MMP-1, IL-10, TNF-α, CCL3, 4E-BP1, LIF, MCP-2, CASP-8, CCL20, STAMBP, and others, spanning a broad set of inflammation-related proteins. Because 87 proteins were tested in 15 patients without correction for multiple comparisons, these results are highly susceptible to false-positive findings and are reported for descriptive screening only, not as evidence that these proteins are associated with injury severity or useful for outcome prediction after TSCI.

Multiple linear regression

Table S6. Multiple linear regression based on the selected quantitative assay biomarkers in peripheral blood against admission injury severity

	Estimate	Standard Error	t value	Pr(>|t|)	
(Intercept)	48.793	11.120	4.388	0.0014	**
IL-6	-0.471	0.603	-0.780	0.4533	
IL-8	-1.739	1.051	-1.654	0.1292	
NfL	0.073	0.099	0.736	0.4785	
GFAP	-0.001	0.001	-0.800	0.4420	

*Signif. codes: 0 <= '***' < 0.001 < '**' < 0.01 < '*' < 0.05*

Residual standard error: 23.3 on 10 degrees of freedom

Multiple R-squared: 0.422, Adjusted R-squared: 0.1908

	Estimate	Standard Error	t value	Pr(>|t|)

F-statistic: 1.825 on 10 and 4 DF, p-value: 0.2006

Table S6. Multiple linear regression of admission injury severity (adjusted AMS) using peripheral blood quantitative assay biomarkers selected from ANOVA. Estimates, standard errors, t-values, and p-values are presented for each predictor. None of the biomarkers reached statistical significance in this model, and the overall model explained a moderate proportion of the variance (Adjusted R^2 = 0.19), indicating limited predictive value with the current sample size.

Table S7. Multiple linear regression based on the selected quantitative assay biomarkers in peripheral blood against the follow-up neurological status

	Estimate	Standard Error	t value	Pr(>|t|)	
(Intercept)	86.287	14.062	6.136	0.0001	***
IL-6	-0.590	0.763	-0.774	0.4570	
IL-8	-2.341	1.329	-1.761	0.1087	
NfL	0.084	0.125	0.676	0.5143	
GFAP	-0.001	0.001	-1.166	0.2707	

*Signif. codes: 0 <= '***' < 0.001 < '**' < 0.01 < '*' < 0.05*

Residual standard error: 29.47 on 10 degrees of freedom

Multiple R-squared: 0.5622, Adjusted R-squared: 0.3871

F-statistic: 3.21 on 10 and 4 DF, p-value: 0.0613

Table S7. Multiple linear regression of follow-up neurological status (adjusted AMS) using peripheral blood quantitative assay biomarkers selected from ANOVA. Estimates, standard errors, t-values, and p-values are presented for each predictor. None of the biomarkers reached statistical significance in this model, and the overall model explained a moderate proportion of the variance (Adjusted R^2 = 0.38), indicating limited predictive value with the current sample size.

Table S8. Multiple linear regression based on the selected Olink panel biomarkers in peripheral blood against admission injury severity

	Estimate	Standard Error	t value	Pr(>|t|)
(Intercept)	809.892			
IL-8	50.681			
VEGFA	-113.677			
MCP-3	-46.907			
GDNF	-63.624			

	Estimate	Standard Error	t value	Pr(>|t|)
uPA	-98.740			
IL-6	9.371			
TRAIL	51.642			
IL-20RA	-134.043			
TSLP	-47.968			
SCF	70.274			
CCL11	30.515			
TNFSF14	82.813			
CCL19	-4.213			
CXCL5	-8.808			

*Signif. codes: 0 <= '***' < 0.001 < '**' < 0.01 < '*' < 0.05*

Residual standard error: NaN on 0 degrees of freedom

Multiple R-squared: 1, Adjusted R-squared: NaN

F-statistic: NaN on 0 and 14 DF, p-value: NaN

Table S8. Multiple linear regression of admission injury severity (adjusted AMS) using peripheral blood Olink panel biomarkers selected from ANOVA. Estimates for each predictor are presented. The model could not be fully evaluated due to zero residual degrees of freedom, resulting in undefined standard errors, adjusted R^2, and F-statistic. While this indicates perfect fit for the included predictors, it reflects overfitting rather than generalizable predictive power, underscoring the need for larger sample sizes for Olink panel models.

Table S9. Multiple linear regression based on the selected Olink panel biomarkers in peripheral blood against the follow-up neurological status

	Estimate	Standard Error	t value	Pr(>|t|)
(Intercept)	565.951			
IL-8	2.382			
VEGFA	7.604			
MCP-3	2.027			
GDNF	239.240			
uPA	-10.072			

	Estimate	Standard Error	t value	Pr(>|t|)
IL-6	1.868			
TRAIL	-28.788			
IL-20RA	74.811			
TSLP	-151.936			
SCF	-40.242			
CCL11	-37.345			
TNFSF14	-12.553			
CCL19	12.276			
CXCL5	-16.157			

*Signif. codes: 0 <= '***' < 0.001 < '**' < 0.01 < '*' < 0.05*

Residual standard error: NaN on 0 degrees of freedom

Multiple R-squared: 1, Adjusted R-squared: NaN

F-statistic: NaN on 0 and 14 DF, p-value: NaN

Table S9. Multiple linear regression of follow-up neurological status (adjusted AMS) using peripheral blood Olink panel biomarkers selected from ANOVA. Estimates for each predictor are presented. The model could not be fully evaluated due to zero residual degrees of freedom, resulting in undefined standard errors, adjusted R^2, and F-statistic. While this indicates perfect fit for the included predictors, it reflects overfitting rather than generalizable predictive power, underscoring the need for larger sample sizes for Olink panel models.

Table S10. Multiple linear regression based on the selected quantitative assay biomarkers in cerebrospinal fluid against the follow-up neurological status

	Estimate	Standard Error	t value	Pr(>|t|)	
(Intercept)	35.336	16.500	2.142	0.1217	
IL-6	-0.549	0.128	-4.284	0.0234	*
IL-8	-0.036	0.025	-1.403	0.2552	
IFN-γ	-62.016	11.938	-5.195	0.0139	*
TNF-α	-62.728	23.137	-2.711	0.0731	.
IL-2	79.139	12.697	6.233	0.0083	**
IL-12	-33.874	7.006	-4.835	0.0169	*

	Estimate	Standard Error	t value	Pr(>|t|)	
IL-4	689.945	135.050	5.109	0.0145	*
IL-10	12.004	13.499	0.889	0.4394	
IL-13	10.415	6.484	1.606	0.2066	
NSE	7.002	1.935	3.618	0.0363	*
GFAP	-1.25×10^{-5}	3.53×10^{-6}	-3.528	0.0387	*

*Signif. codes: 0 <= '***' < 0.001 < '**' < 0.01 < '*' < 0.05*

Residual standard error: 13.49 on 3 degrees of freedom

Multiple R-squared: 0.9725, Adjusted R-squared: 0.8716

F-statistic: 9.638 on 3 and 11 DF, p-value: 0.0438

Table S10. Multiple linear regression of follow-up neurological status (adjusted AMS) using cerebrospinal fluid quantitative assay biomarkers selected from ANOVA. Estimates, standard errors, t-values, and p-values are presented for each predictor. Although several biomarkers reached nominal significance ($p \leq 0.05$) and the model accounted for a large share of the variance (Adjusted $R^2 = 0.872$), these results stem from fitting 11 predictors to 15 observations with only 3 residual degrees of freedom. Such a model is almost certainly overfit, and the apparent significance and high R^2 are expected artifacts of the predictor-to-sample ratio rather than evidence of genuine associations with motor recovery. The coefficients should not be interpreted as biomarker effects.

Table S11. Multiple linear regression based on the selected quantitative assay biomarkers in cerebrospinal fluid against admission injury severity

	Estimate	Standard Error	t value	Pr(>|t|)
(Intercept)	-6.161	31.463	-0.196	0.8573
IL-6	-0.230	0.244	-0.942	0.4158
IL-8	-0.072	0.048	-1.487	0.2338
IFN-γ	-32.954	22.764	-1.448	0.2435
TNF-α	-42.248	44.120	-0.958	0.4089
IL-2	56.412	24.211	2.330	0.1021
IL-12	-15.711	13.359	-1.176	0.3244
IL-4	344.613	257.525	1.338	0.2732
IL-10	4.643	25.742	0.180	0.8684
IL-13	18.314	12.364	1.481	0.2352

	Estimate	Standard Error	t value	Pr(>|t|)
NSE	5.830	3.690	1.580	0.2123
GFAP	-1.35×10^{-5}	6.74×10^{-6}	-2.008	0.1383

*Signif. codes: 0 <= '***' < 0.001 < '**' < 0.01 < '*' < 0.05*

Residual standard error: 25.72 on 3 degrees of freedom

Multiple R-squared: 0.7888, Adjusted R-squared: 0.01424

F-statistic: 1.018 on 3 and 11 DF, p-value: 0.5635

Table S11. Multiple linear regression of admission injury severity (adjusted AMS) using cerebrospinal fluid quantitative assay biomarkers selected from ANOVA. Estimates, standard errors, t-values, and p-values are presented for each predictor. None of the biomarkers reached statistical significance in this model, and the overall model was not statistically significant (F = 1.018 on 3 and 11 DF, p = 0.56; Adjusted R^2 = 0.01), indicating limited predictive value with the current sample size.

Table S12. Multiple linear regression based on the selected Olink panel biomarkers in cerebrospinal fluid against admission injury severity

	Estimate	Standard Error	t value	Pr(>|t|)
(Intercept)	-5,190.255			
IL-8	-1.783			
VEGFA	11.227			
MCP-3	35.669			
CD244	58.288			
OPG	210.586			
LAP-TGF-β-1	-301.663			
uPA	-37.507			
IL-6	-39.955			
MCP-1	20.829			
CXCL11	-41.436			
AXIN1	-0.593			
TRAIL	80.523			
CXCL9	-0.092			
CST5	696.604			

	Estimate	Standard Error	t value	Pr(>|t|)

*Signif. codes: 0 <= '***' < 0.001 < '**' < 0.01 < '*' < 0.05*

Residual standard error: NaN on 0 degrees of freedom

Multiple R-squared: 1, Adjusted R-squared: NaN

F-statistic: NaN on 0 and 14 DF, p-value: NaN

Table S12. Multiple linear regression of admission injury severity (adjusted AMS) using cerebrospinal fluid Olink panel biomarkers selected from ANOVA. Estimates for each predictor are presented. The model could not be fully evaluated due to zero residual degrees of freedom, resulting in undefined standard errors, adjusted R^2, and F-statistic. While this indicates perfect fit for the included predictors, it reflects overfitting rather than generalizable predictive power, underscoring the need for larger sample sizes for Olink panel models.

Table S13. Multiple linear regression based on the selected Olink panel biomarkers in cerebrospinal fluid against the follow-up neurological status

	Estimate	Standard Error	t value	Pr(>|t|)
(Intercept)	-8,514.173			
IL-8	-29.558			
VEGFA	-74.594			
MCP-3	133.276			
CD244	106.047			
OPG	15.008			
LAP-TGF-β-1	-356.245			
uPA	-210.109			
IL-6	-21.313			
MCP-1	39.530			
CXCL11	-116.018			
AXIN1	89.601			
TRAIL	105.893			
CXCL9	38.178			
CST5	2,041.692			

*Signif. codes: 0 <= '***' < 0.001 < '**' < 0.01 < '*' < 0.05*

	Estimate	Standard Error	t value	Pr(>|t|)

Residual standard error: NaN on 0 degrees of freedom

Multiple R-squared: 1, Adjusted R-squared: NaN

F-statistic: NaN on 0 and 14 DF, p-value: NaN

Table S13. Multiple linear regression of follow-up neurological status (adjusted AMS) using cerebrospinal fluid Olink panel biomarkers selected from ANOVA. Estimates for each predictor are presented. The model could not be fully evaluated due to zero residual degrees of freedom, resulting in undefined standard errors, adjusted R^2, and F-statistic. While this indicates perfect fit for the included predictors, it reflects overfitting rather than generalizable predictive power, underscoring the need for larger sample sizes for Olink panel models.

Discussion

Across all eight ANOVA-plus-regression combinations, this supplementary analysis yielded no interpretable findings. The only model that reached statistical significance (follow-up neurological status from CSF quantitative assay biomarkers, Table S10) fitted 11 predictors to 15 observations and is attributable to overfitting rather than to a true signal, while the Olink panel models were saturated (zero residual degrees of freedom) and could not be evaluated at all. Combined with the absence of multiple-comparison correction, none of these results support conclusions regarding admission injury severity or follow-up neurological status. It should further be noted that the univariate screening presented here and the repeated-LASSO analysis of the main study address different questions: the ANOVA tests each biomarker in isolation against AIS grade, whereas LASSO evaluates the biomarkers jointly and penalizes collinear predictors. Divergences between the two approaches, for example IFN-γ in peripheral blood, which did not reach nominal significance in Table S2, and GFAP, which did, are therefore expected, and the two analyses should be read as complementary rather than contradictory. This analysis is therefore presented not as evidence of biomarker associations, but solely to document the rationale for adopting the repeated-LASSO modeling strategy used in the main study, which was chosen specifically to mitigate the overfitting illustrated here.

Table S14. Selection frequency of quantitative assay biomarkers modeling admission injury severity in repeated LASSO models

Biomarker	Blood (%)	CSF (%)
IFN-γ	99.8 (+)	100.0 (−)
IL-8	100.0 (−)	0.0
IL-6	99.8 (−)	0.0
IL-10	0.0	95.6 (−)
GFAP	0.0	2.0 (−)

Biomarker	Blood (%)	CSF (%)
NfL	0.0	2.0 (+)
IL-12	0.5 (+)	0.0

Values indicate the percentage of times each biomarker was selected in 1000 iterations of the LASSO model, stratified by sample type (blood and cerebrospinal fluid (CSF)). Direction (+/-) indicates the sign of the median non-zero coefficient across iterations in which the biomarker was selected.

Table S15. Selection frequency of quantitative assay biomarkers modeling follow-up neurological status in repeated LASSO models

Biomarker	Blood (%)	CSF (%)
IFN-γ	2.9 (+)	100.0 (−)
IL-8	100.0 (−)	0.0
GFAP	95.8 (−)	0.1 (−)
IL-6	95.8 (−)	0.0
IL-10	0.0	0.1 (−)

Values indicate the percentage of times each biomarker was selected in 1000 iterations of the LASSO model, stratified by sample type (blood and cerebrospinal fluid (CSF)). Direction (+/-) indicates the sign of the median non-zero coefficient across iterations in which the biomarker was selected.

Table S16. Selection frequency of Olink panel biomarkers modeling admission injury severity in repeated LASSO models

Biomarker	Blood (%)	CSF (%)
TSLP	100.0 (−)	13.0 (−)
CCL3	2.3 (−)	100.0 (−)
MMP-10	95.5 (+)	0.0
TWEAK	0.0	87.0 (+)
IL-17A	80.6 (−)	0.0
CD8A	21.6 (+)	6.1 (+)
CCL20	21.6 (−)	0.0
CX3CL1	0.0	15.7 (+)
IFN-γ	0.0	15.7 (−)

Biomarker	Blood (%)	CSF (%)
CCL19	0.0	15.6 (+)
FGF-5	0.0	15.6 (+)
CCL28	0.0	15.5 (+)
CDCP1	0.0	15.5 (+)
LIF-R	0.0	13.8 (+)
IL-6	0.0	13.0 (−)
LIF	0.0	12.4 (−)
PD-L1	0.0	9.5 (+)
IL-10RB	0.0	9.1 (+)
IL-12B	0.0	6.1 (−)
TNFSF14	0.0	6.1 (−)
TGF-α	0.0	5.2 (+)
CXCL10	0.0	3.1 (+)
IL-1α	0.0	2.7 (+)
IL-24	2.3 (−)	0.0
IL-2	0.1 (+)	0.0

Values indicate the percentage of times each biomarker was selected in 1000 iterations of the LASSO model, stratified by sample type (blood and cerebrospinal fluid (CSF)). Direction (+/-) indicates the sign of the median non-zero coefficient across iterations in which the biomarker was selected.

Table S17. Selection frequency of Olink panel biomarkers modeling follow-up neurological status in repeated LASSO models

Biomarker	Blood (%)	CSF (%)
CCL20	100.0 (−)	0.0
TSLP	100.0 (−)	0.0
CCL3	0.0	100.0 (−)
IFN-γ	0.0	86.6 (−)
IL-20RA	0.0	86.6 (+)
FGF-5	0.0	83.3 (+)

Biomarker	Blood (%)	CSF (%)
CCL28	0.0	83.2 (+)
CCL4	0.0	83.2 (−)
CD40	0.0	83.2 (−)
CDCP1	0.0	83.2 (+)
CXCL9	0.0	83.2 (−)
IL-12B	0.0	83.2 (−)
CD8A	0.0	77.2 (+)
CX3CL1	0.0	63.0 (+)
TNFB	0.0	54.6 (−)
CXCL10	0.0	51.0 (+)
TGF-α	0.0	45.3 (+)
MCP-4	0.0	22.7 (−)
HGF	15.0 (−)	0.0

Values indicate the percentage of times each biomarker was selected in 1000 iterations of the LASSO model, stratified by sample type (blood and cerebrospinal fluid (CSF)). Direction (+/-) indicates the sign of the median non-zero coefficient across iterations in which the biomarker was selected.

Table S18. Cross-validated predictive performance of repeated LASSO models

Panel	Sample type	Formulation	Median RMSE	RMSE Q25	RMSE Q75
Quantitative assay	Blood	Admission (biomarkers only)	23.1	22.5	23.7
Quantitative assay	CSF	Admission (biomarkers only)	26.0	25.6	26.5
Olink	Blood	Admission (biomarkers only)	22.9	21.9	24.0
Olink	CSF	Admission (biomarkers only)	27.8	27.3	28.4
Quantitative assay	Blood	Follow-up (biomarkers only)	36.6	35.7	37.6
Quantitative assay	CSF	Follow-up (biomarkers only)	34.8	34.2	35.7
Olink	Blood	Follow-up (biomarkers only)	39.4	38.7	40.1

Panel	Sample type	Formulation	Median RMSE	RMSE Q25	RMSE Q75
Olink	CSF	Follow-up (biomarkers only)	32.0	30.4	34.3
Quantitative assay	Blood	Follow-up (biomarkers + admission severity forced in)	25.9	25.4	26.5
Quantitative assay	CSF	Follow-up (biomarkers + admission severity forced in)	24.8	24.1	25.4
Olink	Blood	Follow-up (biomarkers + admission severity forced in)	26.5	26.0	27.1
Olink	CSF	Follow-up (biomarkers + admission severity forced in)	22.7	22.2	23.3
Quantitative assay	Blood	Follow-up (raw outcome, biomarkers + level forced in)	31.7	31.1	32.4
Quantitative assay	CSF	Follow-up (raw outcome, biomarkers + level forced in)	28.0	27.5	28.6
Olink	Blood	Follow-up (raw outcome, biomarkers + level forced in)	31.3	30.1	32.2
Olink	CSF	Follow-up (raw outcome, biomarkers + level forced in)	32.0	31.5	32.7

Median cross-validated root mean squared error (RMSE) at the selected lambda, across 1000 repeated LASSO models. Rows 1-12 (Admission, biomarkers only; Follow-up, biomarkers only; Follow-up, biomarkers + admission severity forced in) are on the level-adjusted percentage AMS scale (0-100). Rows 13-16 (Follow-up, raw outcome, biomarkers + level forced in) are on the raw AMS scale (0-100) instead. Both scales span 0-100, but they are not the same underlying quantity: RMSE is comparable within each block of four rows (same formulation, across the four panels), and across the Admission/Follow-up/Adjusted blocks for a given panel to assess whether forcing in admission severity improves prediction, but should not be compared directly against the final four (Reformulated) rows, which use a different outcome definition. Q25/Q75 denote the interquartile range across the 1000 repetitions.

Table S18. Cross-validated RMSE for all sixteen repeated-LASSO formulations; see main text section 3.4.
